# Avoiding contingent incidents by quadrotors due to one or two propellers failure

**DOI:** 10.1371/journal.pone.0282055

**Published:** 2023-03-03

**Authors:** Kemal Orçun Altınuç, Muhammad Umer Khan, Jamshed Iqbal

**Affiliations:** 1 Department of Mechatronics Engineering, Atilim University, Ankara, Turkey; 2 School of Computer Science, Faculty of Science and Engineering, University of Hull, Hull, United Kingdom; Beijing University of Posts and Telecommunications, CHINA

## Abstract

With the increasing impact of drones in our daily lives, safety issues have become a primary concern. In this study, a novel supervisor-based active fault-tolerant (FT) control system is presented for a rotary-wing quadrotor to maintain its pose in 3D space upon losing one or two propellers. Our approach allows the quadrotor to make controlled movements about a primary axis attached to the body-fixed frame. A multi-loop cascaded control architecture is designed to ensure robustness, stability, reference tracking, and safe landing. The altitude control is performed using a proportional-integral-derivative (PID) controller, whereas linear-quadratic-integral (LQI) and model-predictive-control (MPC) have been investigated for reduced attitude control and their performance is compared based on absolute and mean-squared error. The simulation results affirm that the quadrotor remains in a stable region, successfully performs the reference tracking, and ensures a safe landing while counteracting the effects of propeller(s) failures.

## Introduction

The quadrotors that belong to a unique class of multi-rotor unmanned aerial vehicles (UAVs) have the capability of vertical take-off and landing (VTOL), mainly due to four vertically-aligned rotors. Due to high maneuverability, simple mechanical structure, and low maintenance requirements, they are widely used in military [1], inspection [2], media [3], delivery [4, 5], and surveillance [6] among others. However, several challenges must be addressed before introducing quadrotors to these areas, in particular that of ensuring the safe maneuvering and accomplishment of the given task in case of propeller failure, while ensuring safety.

The quadrotors are associated with a class of under-actuated systems; hence, are considered to have complex control systems to maintain their stability [7, 8]. This can be even more challenging in case of any malfunctioning, such as propeller failure, which could not only lead to the loss of expensive equipment but could also pose a possible threat to the lives and property nearby. Environmental disturbances, hardware and software faults, and other user-related factors can be regarded as the possible causes of failure. Most quadrotors do not have built-in FT capabilities unless special controllers are designed for this purpose to ensure stability in case of propeller(s) failure. Therefore it is important to design FT control systems that can endure potential propeller failures to ensure the safety of the quadrotor and its surroundings.

Lately, a class of FT controllers for quadrotors has been rapidly evolving and presented itself as a promising and effective field of research. The FT control can be used to regain control of the quadrotor when partial loss of thrust generated by the propellers or complete propeller failure occurs. The authors in [9–11] proposed geometric changes in the structure of the quadrotor to compensate against any faulty behavior. The main impetus of this paper comes from the idea to introduce additional stability to help quadrotors in maintaining pose in 3D space and performing safe maneuverings despite losing one propeller or two opposing propellers.

The partial loss of thrust generated by propellers has received the attention of many researchers [12–16]. In [12], the authors utilized a backstepping control approach to achieve trajectory tracking control in case of 50% loss of thrust in propellers. The authors in [13] proposed an incremental sliding mode control (SMC) with a sliding mode disturbance observer to reduce the model dependency of the controller in the presence of partial thrust loss of rotors, disturbances, and model uncertainties. In [14], the issue of loss of effectiveness in one or more propellers was investigated and tested experimentally using gain-scheduled PID control and model-reference adaptive control.

Many researchers have shown keen interest in counteracting the effects of propeller failure to ensure safety [17–29]. In [17], the stabilization of a quadrotor upon complete propeller failure was studied based on a non-singular terminal SMC. The work in [18] introduced an LQR-based attitude controller and a PID controller to stabilize the quadrotor. The authors in [19] proposed a double control loop architecture using a feedback linearization approach to make the quadrotor enter a spin around its vertical axis while maintaining zero angular velocities around the horizontal axis. In a follow-up work [20], the same authors linearized the system around a point with zero roll-and-pitch angles and non-zero yaw angular velocity around the body fixed *z* axis. An $H_{\infty }$ loop-shaping technique was also utilized to control the roll-and-pitch angles, where the outer loop controls the translational movement of the quadrotor with small angle changes on roll-and-pitch angles. An incremental nonlinear dynamic inversion (INDI) attitude controller was investigated in [21] that enabled the quadrotor to achieve any position in 3D space even after the complete failure of a propeller. Backstepping and PD attitude controllers for an emergency landing in case of one propeller failure were discussed in [22] and [23], respectively. The recovery of a quadrotor from major disruptive inputs in case of a complete propeller failure with a cascaded P-PID controller was investigated in [24] where the quadrotor was able to recover after being tossed into the air even after experiencing a propeller failure. In [25], the authors discussed the control of a quadrotor with complete loss of a propeller in high-speed conditions using INDI attitude control and PID position controllers. In [30] and [31], a detailed review is provided on the available hovering recovery strategies in case of a single rotor failure.

In this study, the concept of reduced-attitude control has been utilized [27, 28] to develop an active FT control system. The introduced controller architecture consists of two cascaded controllers: an outer (slower) control loop generates the desired acceleration, and an inner (faster) control loop controls the attitude of the quadrotor in order to attain the desired acceleration. The inner loop is extended further to build a reconfigurable FT control system where a supervisor is able to sense the fault in propellers and accordingly generate an appropriate excitation signal. This signal is responsible for selecting the appropriate controller configuration and switching between the nominal controller and the controller reconfiguration module.

The contributions of this study are summarized as follows:

- A supervisor-based reconfigurable FT control architecture is proposed to perform safe maneuvering in case of propeller(s) failure.
- Hovering conditions for quadrotors in case of propeller(s) failures are derived.
- A cascaded control strategy with PID as a position controller, and LQI and MPC as attitude control techniques are investigated and modified for the implementation.
- The performance of LQI and MPC have been evaluated and compared in terms of absolute and mean-squared error.

The System description and modelling section outline the basic assumptions and system dynamics. The Supervisor-based active FT control system describes the reconfigurable architecture. The Hovering equilibrium conditions upon propeller(s) failure section derives the equilibrium states for failure scenarios. The control strategies for the attitude and altitude of a quadrotor are described in the Controller design section. The Simulation results section presents results obtained for a quadrotor that suffers from propeller failure. The inertias obtained for the custom quadrotor are presented in Parameter estimation for the custom quadrotor. The Conclusion section concludes the paper.

## System description and modelling

The movements of the quadrotor can be defined using translational and rotational motions. The equations of motion help us to better understand the dynamics of a quadrotor by building a reliable model. Through simulations, this model is used to design the attitude and the position controllers. All the variables involved in deriving the dynamic model are defined in Tables 1 and 2.

**Table 1:** Nomenclature.

Parameter	Description
*u*, *v*, *w*	Linear velocity expressed in body-fixed coordinate frame
*p*, *q*, *r*	Angular rates expressed in body-fixed coordinate frame
*X*, *Y*, *Z*	Position of quadrotor in inertial coordinate frame
*ϕ*, *θ*, *ψ*	Roll, Pitch, and Yaw Euler angles
*F*_*i*_	Thrust force produced by *i*^*th*^ rotor in body-fixed coordinate frame
*F*_*total*_	Total motor thrust
*ω*_*i*_	Angular velocity of the *i*^*th*^ rotor
*η*	Primary unit axis in body-fixed frame
*I* ^*B*^	Inertia of the quadrotor body

**Table 2:** Parameters of the quadrotor.

Parameter	Description	Value	Unit
*m*	Mass of quadrotor	1.0543	kg
*l*	Arm length	0.2258	m
*g*	Gravitational acceleration	9.81	m/s^2^
*k*_*T*_	Propeller thrust coefficient	6.41*e*−6	Ns^2^/rad^2^
*k*_*d*_	Propeller drag coefficient	1.69*e*−2	Nm/N
*γ*	Quadrotor drag coefficient	2.75*e*−3	Nms/rad
*I* ^*T*^	Total inertia of the quadrotor	$\left[\begin{array}{ccc} 8.5e-3 & -4.04e-4 & 2.33e-5\\ -4.04e-4 & 8.9e-3 & -1.73e-4\\ 2.33e-5 & -1.73e-4 & 1.51e-2 \end{array}\right]$	kg-m^2^
*I* ^*p*^	Propeller inertia	4.125*e*−5	kg-m^2^

To derive the equations of motion of the quadrotor, the following assumptions are made:

**Assumption 1:** The quadrotor is rigid and symmetric, and its center of mass is located at the center of the chassis.
**Assumption 2:** Thrust and drag are proportional to the square of the speed of propellers.
**Assumption 3:** Translational drag is neglected as the quadrotor is moving at a relatively slow speed.
**Assumption 4:** Rotational drag is assumed to oppose the yaw rate only.

**Fig 1:**
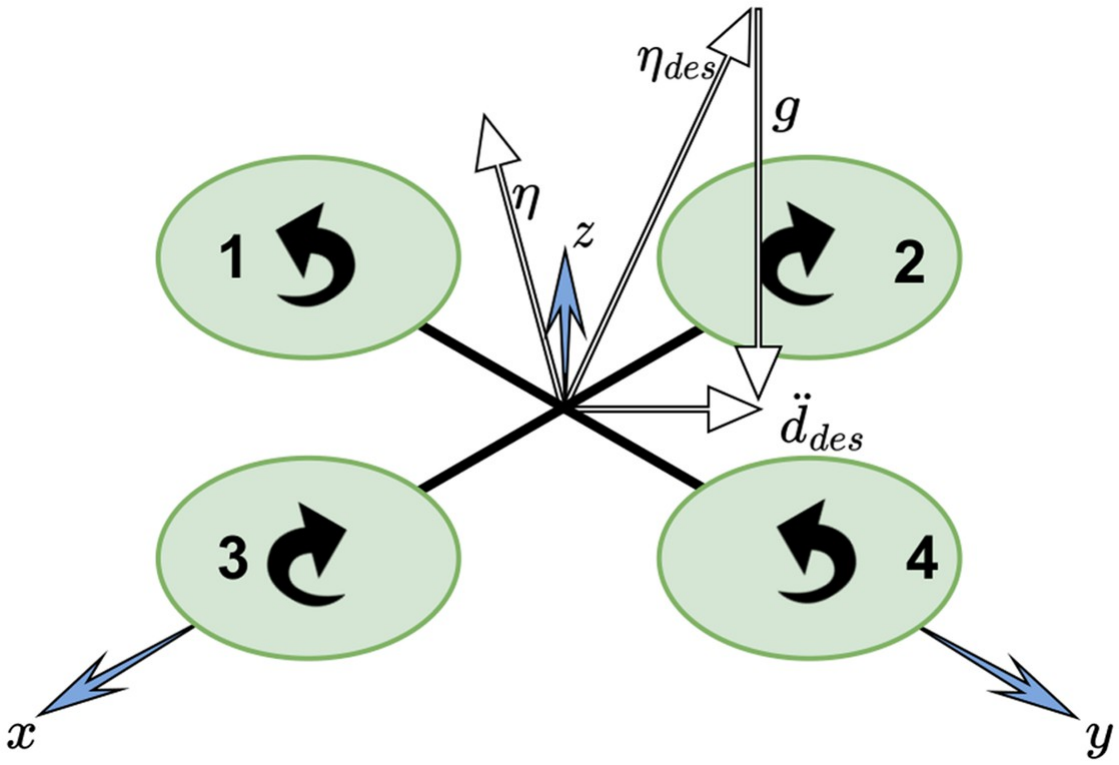
Six degrees of freedom quadrotor model.

The torque (*τ*) affected along the *x*, *y*, and *z* axes on the body-fixed frame (Fig 1) of the quadrotor are expressed as:

$$\begin{array}{c} \left[\begin{array}{c} \tau_{x}\\ \tau_{y}\\ \tau_{z} \end{array}]=[\begin{array}{c} \left(F_{2}-F_{4}\right)l+\tau_{d_{x}}\\ \left(F_{3}-F_{1}\right)l+\tau_{d_{y}}\\ \tau_{1}+\tau_{2}+\tau_{3}+\tau_{4}+\tau_{d_{z}} \end{array}\right] \end{array}$$ (1)

where $\tau_{d}=\left(\tau_{d_{x}},\tau_{d_{y}},\tau_{d_{z}}\right)$ introduces the effect of drag torque. In [27], the effect of drag is assumed only along the *z* axis and is represented as:

$$\begin{array}{c} \tau_{d}=\left(0,0,-\gamma r\right) \end{array}$$ (2)

The reaction torque by the *i*^*th*^ propeller of the quadrotor is generated as:

$$\begin{array}{c} \tau_{i}={\left(-1\right)}^{i+1}\;k_{d}\;F_{i} \end{array}$$ (3)

where *F*_*i*_ represents the thrust force generated by the *i*^*th*^ propeller and represented as:

$$\begin{array}{c} F_{i}=k_{T}\omega_{i}^{2} \end{array}$$ (4)

The nonlinear mathematical model of the 6-DOF quadrotor (Fig 1) is derived using the Newton-Euler method and presented in Eqs (5)–(8). The relationship between the body-fixed velocity and the inertial frame velocity is obtained using:

$$\begin{array}{c} \begin{array}{cc} \left[\begin{array}{c} \dot{X}\\ \dot{Y}\\ \dot{Z} \end{array}\right] & =[\begin{array}{ccc} c\psi \;c\theta & c\theta \;s\psi & -s\theta \\ c\psi \;s\phi \;s\theta -c\phi \;s\psi & c\phi \;c\psi \;+\;s\phi \;s\psi \;s\theta & c\theta \;s\phi \\ s\phi \;s\psi +c\phi \;c\psi \;s\theta & c\phi \;s\psi \;s\theta -c\psi \;s\phi & c\phi \;c\theta \end{array}]\;[\begin{array}{c} u\\ v\\ w \end{array}] \end{array} \end{array}$$ (5)

where *c* and *s* represent the trigonometric cosine and sine functions, respectively. Using the transformation matrix, the angular velocities in both frames are associated as:

$$\begin{array}{c} \left[\begin{array}{c} \dot{\phi }\\ \dot{\theta }\\ \dot{\psi } \end{array}]=[\begin{array}{ccc} 1 & s\phi t\theta & c\phi t\theta \\ 0 & c\phi & -s\phi \\ 0 & \frac{s\phi }{c\theta } & \frac{c\phi }{c\theta } \end{array}]\;[\begin{array}{c} p\\ q\\ r \end{array}\right] \end{array}$$ (6)

where *t* represents the trigonometric tangent function. The linear accelerations in the body-fixed frame of the quadrotor can be defined as:

$$\begin{array}{c} \left[\begin{array}{c} \dot{u}\\ \dot{v}\\ \dot{w} \end{array}]=[\begin{array}{c} rv-qw-gs\theta \\ pw-ru+gc\theta s\phi \\ -qu+pv+c\phi c\theta -\frac{F_{1}+F_{2}+F_{3}+F_{4}}{m} \end{array}\right] \end{array}$$ (7)

The complete dynamic model of the quadrotor is expressed as:

$$\begin{array}{c} \left[\begin{array}{c} \dot{p}I_{xx}^{B}\\ \dot{q}I_{yy}^{B}\\ \dot{r}I_{zz}^{B} \end{array}]+[\begin{array}{c} \left(I_{zz}^{T}-I_{yy}^{T}\right)qr+I_{zz}^{T}q\left(w_{1}+w_{2}+w_{3}+w_{4}\right)\\ -\left(I_{zz}^{T}-I_{xx}^{T}\right)pr-I_{zz}^{T}p\left(w_{1}+w_{2}+w_{3}+w_{4}\right)\\ -k_{T}k_{d}\left({w_{1}}^{2}-{w_{2}}^{2}+{w_{3}}^{2}-{w_{4}}^{2}\right)+\gamma r \end{array}]=[\begin{array}{c} \tau_{x}\\ \tau_{y}\\ \tau_{z} \end{array}\right] \end{array}$$ (8)

where *I*^*T*^ = *I*^*B*^ + 4*I*^*P*^. *I* is the moment of inertia tensor, whereas superscripts *B* and *P* represent the moment of inertia for the quadrotor body and propellers, respectively, and *T* represents the total inertia of the quadrotor.

From Eqs (5)–(8), it can be observed that the translational motion is dependent on the rotational motion, but not vice versa. Eq 9 describes the translational dynamics in the inertial frame as:

$$\begin{array}{c} \left[\begin{array}{c} \ddot{X}\\ \ddot{Y}\\ \ddot{Z} \end{array}]=[\begin{array}{c} c\phi s\theta c\psi +s\phi s\psi \\ c\phi s\theta s\psi -c\phi c\psi \\ c\phi c\theta \end{array}]\frac{F_{total}}{m}+[\begin{array}{c} 0\\ 0\\ -g \end{array}\right] \end{array}$$ (9)

where *g* represents the gravitational constant.

### Reduced-attitude

Quadrotors are considered under-actuated systems as only 4 inputs are available to control their rotational and translational movements in 3D space. In case of a propeller failure, the quadrotor loses one degree of freedom; hence, it is not possible to fully control its movements. While the *ϕ* and *θ* angles are essential to keep the quadrotor stable, these angles also affect position control. The approach adopted here is to abandon the idea of full attitude control, and, instead, control *ϕ*, *θ*, and the altitude of the quadrotor. A concept also referred to as ‘reduced-attitude’ [32]. The reduced-attitude can be expressed as a unit vector motionless in the inertial frame as:

$$\begin{array}{c} \dot{\eta }=-w^{B}\times \eta \end{array}$$ (10)

where *w*^*B*^ = (*p*, *q*, *r*) represents the angular velocity in the body-fixed frame.

## Supervisor-based active FT control system

**Fig 2:**
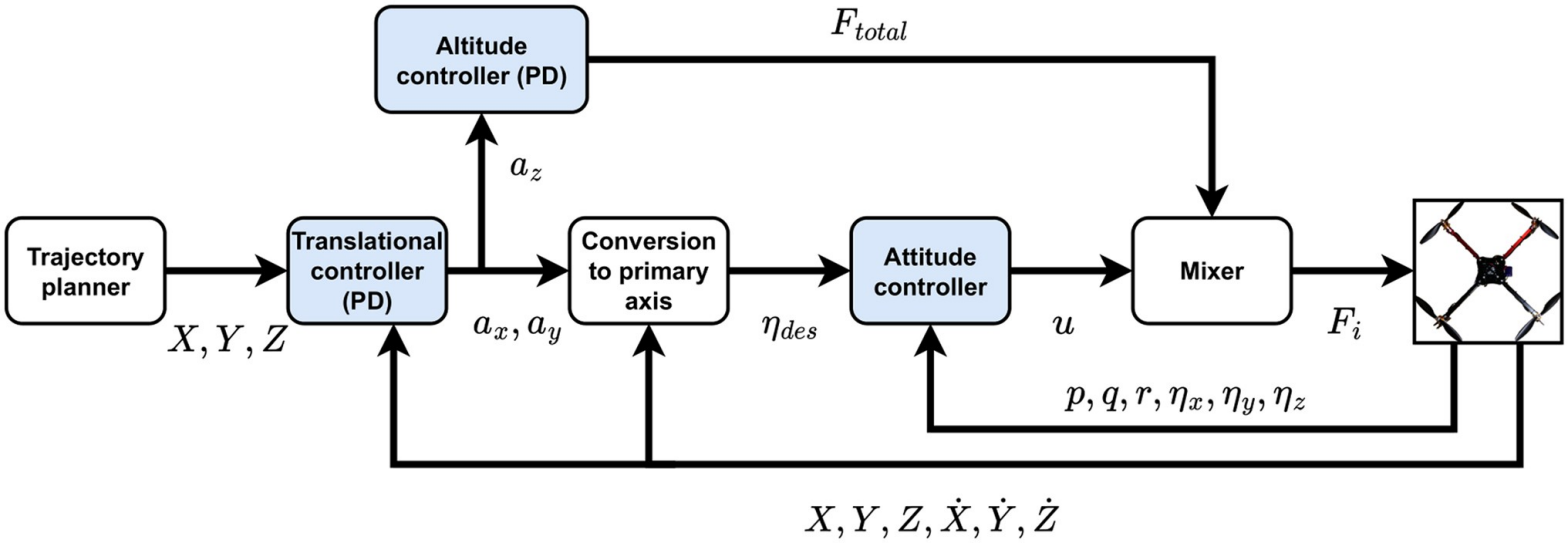
Cascaded control architecture in case of propeller failure.

The proposed approach deals with the quadrotor’s stability control problem in case of propeller(s) failure through cascaded control, as shown in Fig 2. The inner (slower) loop regulates the reduced-attitude of the quadrotor, while the outer (faster) loop regulates the position. The inner reduced-attitude loop is formed using LQI or MPC; whereas, the outer loop regulates the position of the quadrotor through a PID controller.

**Fig 3:**
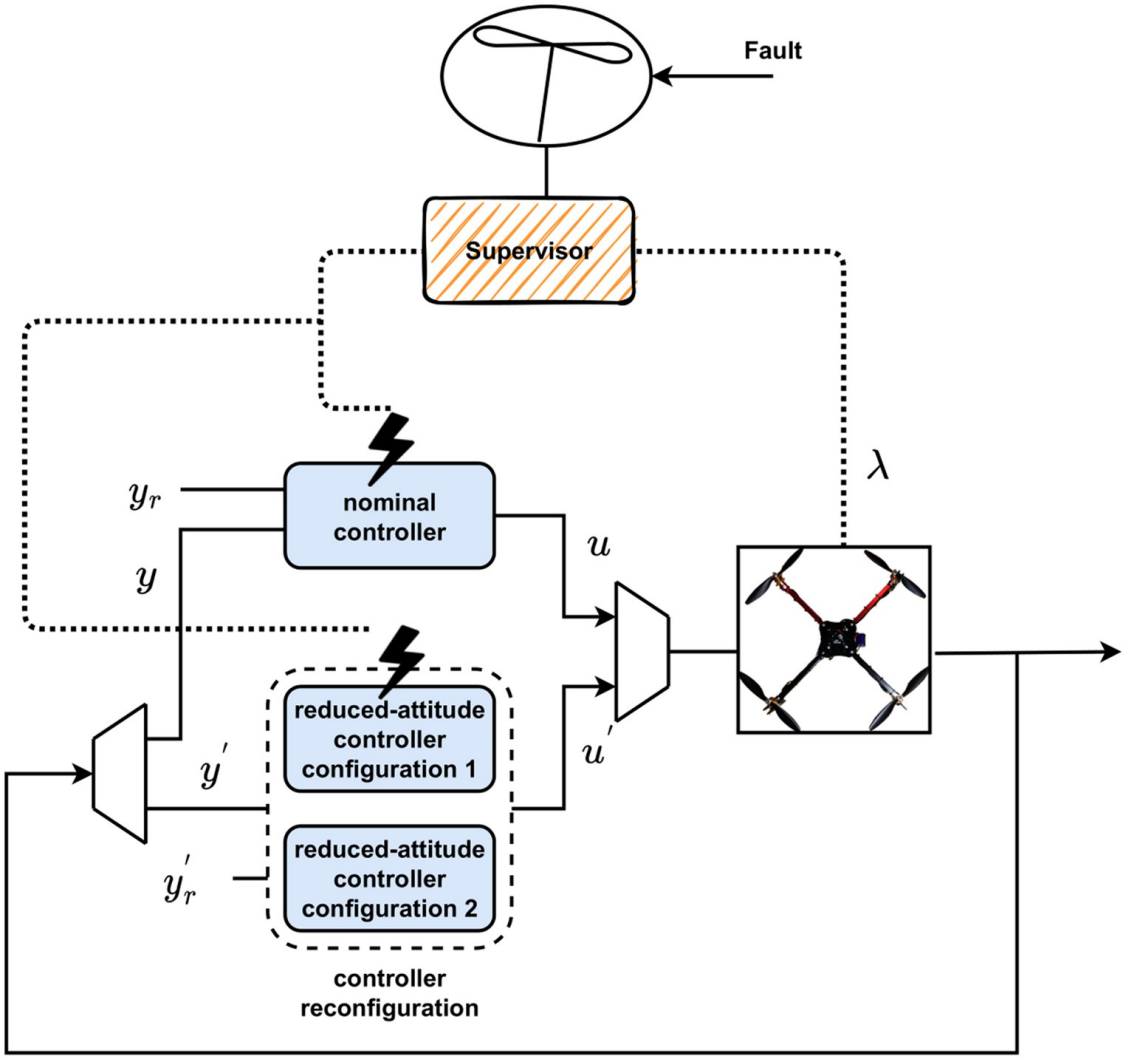
Supervisor-based controller reconfigurable architecture.

To counteract the propeller failure, a supervisor-based controller reconfigurable architecture is proposed in Fig 3. The coefficient λ_*i*_ is introduced to track the status of each propeller as either fully functional (λ_*i*_ = 1) or complete loss (λ_*i*_ = 0). The supervisor module is able to sense the fault introduced to the quadrotor as propeller failure; hence, generates an appropriate excitation signal. Based on the timing and nature (one propeller, two propellers) of the excitation signal, a particular controller configuration is chosen. The proposed controller configuration is easily extendable to any *n* events.

## Hovering equilibrium conditions upon propeller(s) failure

Due to propeller(s) failure, the quadrotor loses its state of static equilibrium as other propellers are still generating non-zero torques. The strategy here is to sacrifice the yaw control, and control the position of the quadrotor through roll-and-pitch instead; yet, this sacrifice could cause undesired spinning of the quadrotor around its body-fixed *z* axis. The primary axis (*η*) expressed in the body-fixed frame (Fig 1) can be used to control the position of the quadrotor independently from the yaw angle. This axis can be thought of as the quadrotor’s thrust direction averaged over one rotation. Using *η* for attitude control makes the overall system more intuitive since there are no *ϕ* and *θ* angles provided to the system.

### Hovering equilibrium state

For a quadrotor that experiences propeller failure, a new equilibrium state is determined. A bar is placed over the variables to define the constants around the periodic solution. The objective is to determine a periodic solution for a primary axis, $\bar{\eta }=\left({\bar{\eta }}_{x},{\bar{\eta }}_{y},{\bar{\eta }}_{z}\right)$, which is attached to the quadrotor’s body-fixed frame. The quadrotor rotates around the primary axis with a constant angular velocity ${\bar{w}}^{B}$. Following the cross product property, it is required that $\bar{\eta }$ should be parallel to the angular velocity ${\bar{w}}^{B}$, as shown in Eq (10), so that:

$$\begin{array}{c} \dot{\bar{\eta }}=0 \end{array}$$ (11)

Since ${\bar{\eta }}_{x}$ is fixed with respect to the body axis of the quadrotor, another requirement for the hover equilibrium is defined as:

$$\begin{array}{c} \bar{\eta }=\epsilon \bar{w} \end{array}$$ (12)

As $\bar{\eta }$ is defined as a unit vector:

$$\begin{array}{c} ||\;\bar{\eta }\;||=||\;\epsilon \bar{w}\;\left|\right| \end{array}$$ (13)

where *ϵ* is defined as a constant.

#### Case I: One propeller failure

In this scenario, it is considered that the 4^th^ propeller of the quadrotor stops working; hence, the other three propellers must generate the desired thrust. To prevent the quadrotor from flipping over, an intuitive solution is to have two opposing propellers generate equal thrust.

$$\begin{array}{c} {\bar{F}}_{1}={\bar{F}}_{3} \end{array}$$ (14)

The tuning factor (*ρ*), defined as a ratio between *F*_1_ and *F*_2_, can be optimized depending on the objectives, such as minimum power consumption or minimum yaw rate.

$$\begin{array}{c} \rho ={\bar{F}}_{2}/{\bar{F}}_{1} \end{array}$$ (15)

Eqs (8), (11)–(15) are solved for the unknowns ${\bar{\eta }}_{x}$, ${\bar{\eta }}_{y}$, ${\bar{\eta }}_{z}$, $\bar{p},\bar{q},\bar{r}$, $∊,{\bar{F}}_{1},{\bar{F}}_{2},$ and ${\bar{F}}_{3}$ with the angular acceleration ${\dot{w}}^{B}$ set to zero to find the hovering equilibrium.

#### Case II: Two opposite propellers’ failure

Without the loss of generality, it is assumed that propellers four and two have stopped working completely.

$$\begin{array}{c} {\bar{F}}_{4,2}={\bar{\tau }}_{4,2}=0 \end{array}$$ (16)

Since the quadrotor is missing two opposite propellers, the other two propellers must generate the desired thrust. To prevent the quadrotor from flipping over, an intuitive solution is to have two opposing propellers generate equal thrust to maintain stability.

$$\begin{array}{c} {\bar{F}}_{1}={\bar{F}}_{3} \end{array}$$ (17)

To find the hovering equilibrium in case of two propellers’ failure, *ρ* also becomes zero.

$$\begin{array}{c} \rho ={\bar{F}}_{2}/{\bar{F}}_{1}=0 \end{array}$$ (18)

Eqs (8), (11)–(15), (17) and (18) are solved for the unknowns ${\bar{\eta }}_{x},{\bar{\eta }}_{y},{\bar{\eta }}_{z}$, $\bar{p},\bar{q},\bar{r}$, $∊,{\bar{F}}_{1}$, and ${\bar{F}}_{3}$ with the angular acceleration ${\dot{w}}^{B}$ set to zero to find the hover equilibrium.

## Controller design

The altitude of a quadrotor is controlled using a PID control, whereas LQI and MPC are investigated to perform the attitude control.

### Position controller

The PID controller regulates the quadrotor’s acceleration defined in the inertial frame, and, consequently, the position of the vehicle. The deviation from the desired point in the inertial frame is defined as *d*, and the rate of deviation is represented as $\dot{d}$. The goal of the position controller is to represent this deviation using a second-order system with natural frequency *ω*_*n*_, damping ratio *ξ*, and desired acceleration ${\ddot{d}}_{des}$, and to control the behavior using:

$$\begin{array}{c} {\ddot{d}}_{des}+2\xi w_{n}\dot{d}+{\omega_{n}}^{2}d=0 \end{array}$$ (19)

As discussed earlier, the position of the quadrotor can be controlled by regulating the body-fixed primary axis *η*. For this purpose, the desired acceleration can be defined by introducing *η*_*des*_:

$$\begin{array}{c} {\ddot{d}}_{des}=\left(\frac{{\bar{\eta }}_{z}}{m}\;F_{total}\right)\;\eta_{des}+g \end{array}$$ (20)

By manipulating the total thrust *F*_*total*_ and the desired primary axis *η*_*des*_, the quadrotor’s desired acceleration and, hence, the position can be defined.

It is almost true for all controllers that a certain criterion is defined for acceptable performance. In this work, we used the decay ratio as a performance criterion as it provides a better picture of the set point response, even in the presence of disturbance. For controller tuning, the performance specification is defined to achieve a quarter-way decay. The controller tuning started with an open-loop test for which the initial guess is obtained using Cohen-Coon [33]. As a standard procedure, the proportional gain is introduced first followed by integral action. Lastly, the derivative is added to improve the stability of the overall system. Instead of making an attempt at trial-and-error tuning, which may or may not result in improved performance, we adopted a more organized procedure for controller tuning defining the relation between the period of oscillation and integral time. For a detailed description of the tuning process with the help of a flow chart, the reader should refer to [33].

### Reduced-attitude controller

Under normal circumstances, the attitude controller allows controlling the full attitude of the quadrotor. However, in case of propeller(s) failure, unbalance in the propeller drag makes the quadrotor spin around its own vertical axis. As full attitude control is no more possible at this stage, the reduced-attitude approach sacrifices the yaw dynamics to ensure overall stability. The controllability of the quadrotor’s reduced-attitude is investigated by linearizing the system near the hovering equilibrium solution by utilizing the time-invariant nature of the hover solution.

The reduced-attitude of the quadrotor is introduced as the state vector *s* = (*p*, *q*, *η*_*x*_, *η*_*y*_). The aim of the reduced-attitude controller is to regulate *η* to *η*_*des*_ while keeping $\dot{\eta }$ fixed at zero. The deviation of the state vector *s* from the hovering equilibrium is expressed by

$$\begin{array}{c} \tilde{s}=s-\bar{s} \end{array}$$ (21)

where $\bar{s}$ is the hovering equilibrium state. By linearizing the system around the equilibrium states:

$$\begin{array}{c} \dot{\tilde{s}}=A\tilde{s}+Bu \end{array}$$ (22)

For the failure scenarios, the control input *B* matrix is modified such that the *j*^*th*^ column of the matrix is replaced by the very same column times a factor lambda that varies from zero (complete loss) to one (fully operational). For one propeller failure, we will have two control inputs; hence λ_1_ and λ_2_ are equal to 1. For two opposing propellers failure, only one control input is available; hence, λ_1_ = 1 and λ_2_ = 0

$$\begin{array}{c} \dot{\tilde{s}}=[\begin{array}{cccc} 0 & \bar{c} & 0 & 0\\ -\bar{c} & 0 & 0\\ 0 & -{\bar{\eta }}_{z} & 0 & \bar{r}\\ {\bar{\eta }}_{z} & 0 & -\bar{r} & 0 \end{array}]\tilde{s}+[\begin{array}{ccccc} b_{11} & \cdots & b_{1j}\lambda_{j} & \cdots & b_{1N}\\ \vdots & \cdots & \vdots & \cdots & \vdots \\ b_{l1} & \cdots & b_{lj}\lambda_{j} & \cdots & b_{lN}\\ \vdots & \cdots & \vdots & \cdots & \vdots \\ b_{p1} & \cdots & b_{pj}\lambda_{j} & \cdots & b_{pN} \end{array}][\begin{array}{c} u_{1}\\ \vdots \\ u_{j}\\ \vdots \\ u_{N} \end{array}] \end{array}$$ (23)

where $\bar{r}$ is the yaw rate equilibrium, *p* specifies the number of states, *N* specifies the number of control inputs, and $\bar{c}$ is the coupling constant defined as:

$$\begin{array}{c} \bar{c}=\frac{I_{xx}^{T}-I_{zz}^{T}}{I_{xx}^{B}}\;\bar{r}-\frac{I_{zz}^{p}}{I_{xx}^{B}}\;\left(\bar{w_{1}}+\bar{w_{2}}+\bar{w_{3}}+\bar{w_{4}}\right) \end{array}$$ (24)

For one propeller failure, only two free input variables are available to control the attitude of the quadrotor since one input variable is used to control the altitude. The deviations of the motor thrusts from the hovering equilibrium motor forces are calculated as:

$$\begin{array}{c} \left[\begin{array}{c} u_{1}\\ u_{2} \end{array}]=[\begin{array}{c} \left(F_{3}-{\bar{F}}_{3}\right)-\left(F_{1}-{\bar{F}}_{1}\right)\\ \left(F_{2}-{\bar{F}}_{2}\right) \end{array}\right] \end{array}$$ (25)

In the scenario where two propellers are functioning, only one free input variable is available to control the attitude of the quadrotor. The deviations of the motor thrusts from the hovering equilibrium motor forces are introduced as:

$$\begin{array}{c} u_{1}=\left(F_{3}-{\bar{F}}_{3}\right)-\left(F_{1}-{\bar{F}}_{1}\right) \end{array}$$ (26)

The control input(s) for both cases can be either generated using LQI or MPC.

#### Linear quadratic integral

In a cascaded control strategy, LQI is introduced as a candidate to perform attitude control of a quadrotor. The control input *u* and structure of input matrix *B* varies from case to case. The integral actions on the error of *η*_*x*_ and *η*_*y*_, defined as a state vector $\tilde{z}$, are augmented to $\tilde{s}$ to obtain augmented state vector $\chi ={\left[\tilde{s},\tilde{z}\right]}^{T}$. The control input *u* around the operating point is calculated according to

$$\begin{array}{c} u=-[\begin{array}{cc} K_{1} & K_{2} \end{array}][\begin{array}{c} \tilde{s}\\ \tilde{z} \end{array}] \end{array}$$ (27)

where $\tilde{z}$ is the integral of the tracking error vector.

The controller gain $K=[\begin{array}{cc} K_{1} & K_{2} \end{array}]$ is obtained by minimizing the given cost function:

$$\begin{array}{c} J=\int_{0}^{\infty }\left(\chi^{T}Q\chi +u^{T}Ru\right)\;dt \end{array}$$ (28)

where weight matrices *Q* and *R* are non-negative symmetric and positive definite for the states and input, respectively. Regardless of the values of *Q* and *R* matrices, the cost function has a unique minimum that can be obtained by solving Algebraic Riccati Equation (ARE). The parameters *Q* and *R* are used to penalize the state variables and the control signal. A higher value is chosen to increase the penalty of these signals. According to a general understanding, a large value means that with less energy a struggle is made to stabilize the system. Contrarily, choosing a small value indicates that no penalty is applied or that the states and the control input or of less importance.

In this work, we used the method defined by Bryson and Ho [34] to determine the initial value for *Q*. The matrix *Q* is a diagonal matrix whose entries correspond to the inverse of the square of maximum deviation *m* i.e., $q_{ii}=\frac{1}{m_{i}^{2}}$. Since there is a trade-off between *Q* and *R*, we just kept the *R* matrix fixed at *I* and made changes to the *Q* matrix.

#### Model predictive control

The model predictive controller, as a reduced-attitude control, is responsible for deriving the quadrotor to newly determined hovering equilibrium points. This process is performed by minimizing the performance index of the optimization problem. The manipulated variable is computed by solving the quadratic programming (QP) optimization.

The optimization functions for the MPC are designed based on the following discrete form of the system [35]

$$\begin{array}{c} \begin{array}{cc} \tilde{s}(k+1) & =f^{k}(\tilde{s}\left(k\right),u\left(k\right))T_{s}+\tilde{s}(k)\\ y(k) & =C\tilde{s}(k) \end{array} \end{array}$$ (29)

where *s* is a reduced-attitude state vector of the quadrotor, and *f*^*k*^ represents the gradient of the system’s state change at instance *k* obtained from the system model.

The first cost function is defined with the objective to minimize the deviation between the outputs and the given references.

$$\begin{array}{c} J_{1}={∥Y(k)-R(k)∥}_{ϒ}^{2} \end{array}$$ (30)

where ϒ is a positive diagonal matrix that is employed for adjusting the tracking performance.

A quadratic positive-definite cost function is introduced as a second performance index to ensure closed-loop Lyapunov stability.

$$\begin{array}{c} J_{2}=\tilde{s}{∥(k+p|k)∥}_{P}^{2} \end{array}$$ (31)

where *P* is a symmetric positive-definite matrix that is used to tune penalties.

MPC has the capability to effectively solve multi-objective optimization problems. Therefore, a composite objective function as a combination of both cost functions is introduced to solve an optimal control problem

$$\begin{array}{c} J_{mpc}\left(\tilde{s}\left(k\right),U\left(k\right)\right)=J_{1}+J_{2} \end{array}$$ (32)

The performance of the MPC depends upon the sampling time *T*_*s*_, prediction horizon *p*, and control horizon *m*.

## Simulation results

The development of a control system has various stages; control system design, control law design, simulation, and testing. Since real-time testing is time-consuming and costly, requiring a safe test environment, especially for quadrotors with propeller failures, simulations are used to develop and test the performance and behavior. Once the performance goals are satisfied and the control structure is verified, the designed controller can be tested in actual settings.

### Simulation framework and scenario

The developed FT control scheme is tested and implemented in Simulink and MATLAB on an Intel Core i7-1270PE processor with a clock speed of up to 4.50GHz. The scenario for testing the performance of the quadrotor is defined in the following:

- The quadrotor is hovering at an altitude of 2m with four functioning propellers. At the time *t* = 1s, propeller failure occurs.
- Once the hovering state is ensured, the quadrotor is intended to commence travel of 2m on the *x* axis at *t* = 1s and, afterward descend to 0.5m at *t* = 16*s*.
- Throughout the simulation, it is desired to maintain the quadrotor’s *y* position at 0m.

### Case I: One propeller failure

The hovering equilibrium conditions for one propeller failure are calculated as detailed in the earlier section. The equilibrium conditions are found as: ${\bar{F}}_{1}={\bar{F}}_{3}=2.04\text{N}$, ${\bar{F}}_{2}=1.02\text{N}$, ${\bar{w}}^{B}=\left(0,\;5.36,\;18.8\right)\text{rad/s}$, $\bar{\eta }=\left(0,0.2744,0.9616\right)$.

**Fig 4:**
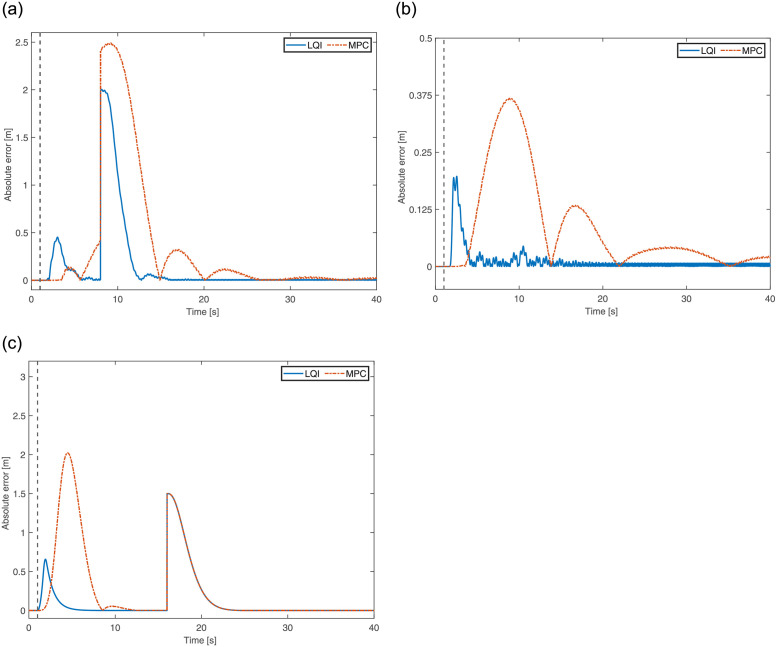
3D position error of the quadrotor for One propeller failure. **(a)** Error in X-axis. **(b)** Error in Y-axis. **(c)** Error in Z-axis.

The quadrotor is initially positioned at (0, 0, 2)m in 3D space with no orientation applied. The absolute position error along *x*, *y* and *z* axis are illustrated in Fig 4a–4c using LQI and MPC. According to the scenario, a fault in the form of a propeller failure at *t* = 1s, a step change in the reference input *X* at *t* = 8s, and a drop in reference altitude to 0.5m at *t* = 16s are applied. The rise in the altitude of the quadrotor is due to the fact that the 4^th^ propeller is disabled at *t* = 1*s*, as shown in Fig 4c. It is assumed that using certain failure diagnosis tools, the information regarding the failure is made available in real time, hence triggering the controller. As is evident from Fig 4c that both controllers, LQI and MPC, perform well to ensure the proper referencing of both the input trajectory and stable hovering. But, the MPC exhibits slightly higher amplitude peaks that lead to some transient movements. Evaluating the performance of both controllers, LQI and MPC, using mean-squared error also supports this argument as the former attains 0.1385m and the latter obtains 0.5266m. Therefore, in terms of mean-squared error, LQI has an edge over MPC.

**Fig 5:**
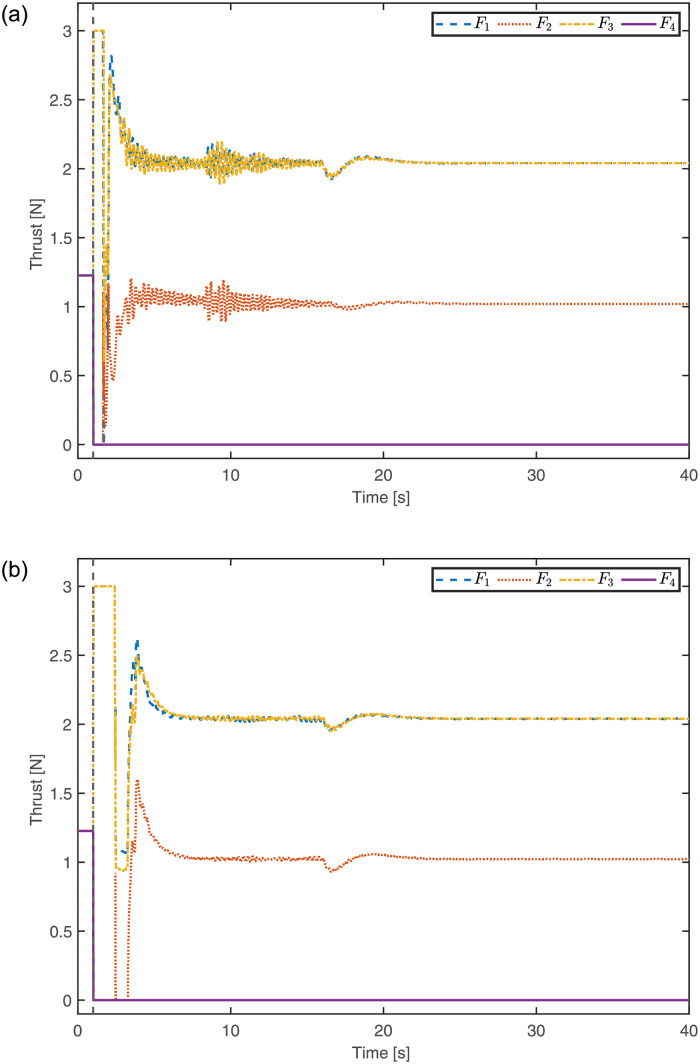
Motor thrusts of the quadrotor in case of One propeller failure. **(a)** Motor thrusts computed using LQI. **(b)** Motor thrusts computed using MPC.

The motor thrusts computed for all propellers using LQI and MPC are also recorded and shown in Fig 5a and 5b. Due to the 4^th^ propeller failure at *t* = 1s, the quadrotor tries to achieve new hovering equilibrium conditions. It can be seen that both controllers effectively attain the equilibrium conditions very promptly. Moreover, both controllers are able to maintain the equilibrium conditions even in the presence of reference input change applied in altitude at *t* = 16s. According to the equilibrium conditions defined, *F*_1_ and *F*_3_ are assumed to experience the same thrust.

**Fig 6:**
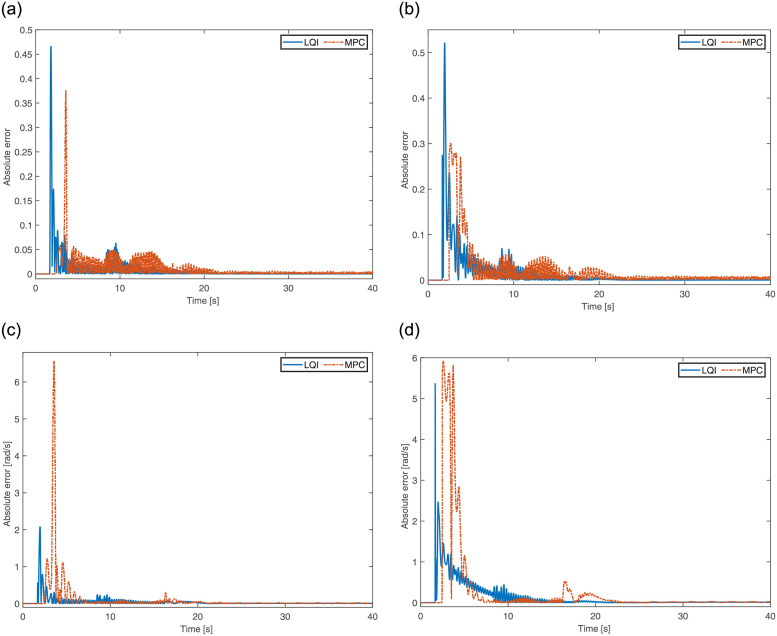
Reduced attitude error of the quadrotor for One propeller failure. **(a)** Absolute error in primary axis for *η_x_*. **(b)** Absolute error in primary axis for *η_y_*. **(c)** Absolute error in angular velocity for *p*. **(d)** Absolute error in angular velocity for *q*.

Initially, the quadrotor uses two opposing propellers to gain ample angular velocity around the body-fixed *Z* axis before the activation of the 2^nd^ propeller. The absolute error for the reduced-altitude states using LQI and MPC are plotted in Fig 6. Due to propeller failure at *t* = 1s, the quadrotor becomes unstable and starts spinning around its own axis. The controllers ensure that spinning around the yaw is controlled in order to avoid crashing. As the quadrotor gains enough angular momentum at around *t* = 2.1s, the reduced attitude controller comes into play to drive the quadrotor states to the hovering equilibrium solution. The transients appear at *t* = 8s as the change in the *X* reference input is applied. After some adjustments, the quadrotor regains its hovering equilibrium before being disturbed again at *t* = 16s with a drop in the altitude. It is observed that both controllers perform well and the quadrotor successfully achieves its hovering equilibrium solution. As observed earlier, MPC has introduced a bit higher peaks as compared to LQI, for *p* and *q* in particular.

### Case II: Two opposing propellers’ failure

The hovering equilibrium conditions for Two opposite propellers’ failure are obtained as: ${\bar{F}}_{1}={\bar{F}}_{3}=2.45\text{N}$, ${\bar{w}}^{B}=\left(0,\;0,\;30.1435\right)\text{rad/s}$, $\bar{\eta }=\left(0,\;0,\;1\right)$.

**Fig 7:**
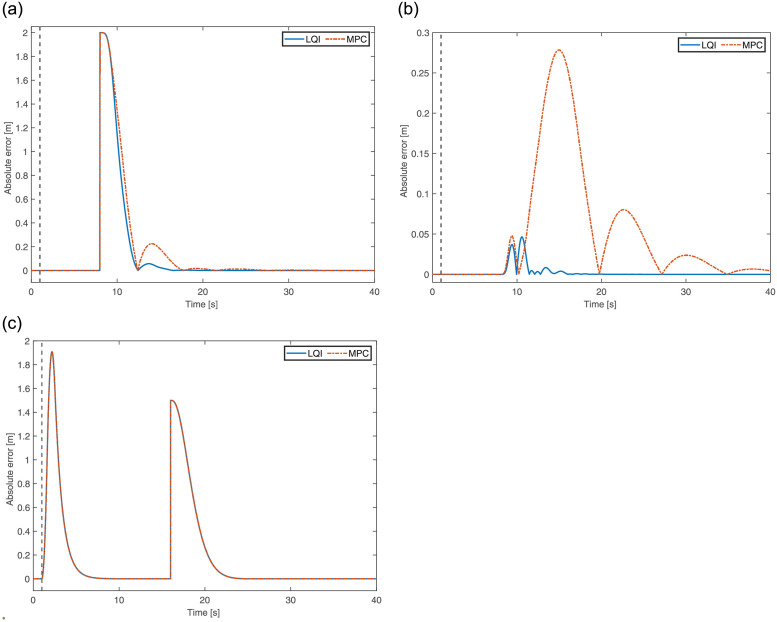
3D position error of the quadrotor for Two opposing propellers’ failure. **(a)** Error in X-axis. **(b)** Error in Y-axis. **(c)** Error in Z-axis.

The scenario and test conditions are kept the same as in case I; hence, the quadrotor starts from the hovering state at an altitude of 2m. The absolute position error plots of the quadrotor in 3D using LQI and MPC are shown in Fig 7. The fault in the form of two propellers failure is introduced at *t* = 1s. As shown in Fig 7a, a step change in the reference input *X* is applied at *t* = 8s, whereas the reference altitude is dropped to 0.5m at *t* = 16s, as shown in Fig 7c. There is no reference input provided along the *y*-axis (Fig 7b). With the change in the reference inputs, both controllers act promptly to re-attain the hovering state by experiencing a small overshoot. The first transient in Fig 7c appears due to the failure of propellers 2 and 4. In a small time period, the controllers ensure that the quadrotor re-attains the hovering equilibrium solution. By comparing LQI and MPC, it can be observed that both controllers exhibit similar responses, except for *y* where the latter generates slightly higher transients. Evaluating their performance using mean-squared error also supports this argument as LQI attains 0.2978m and MPC obtains 0.3462m. Therefore, in terms of performance, LQI holds slight supremacy over MPC.

**Fig 8:**
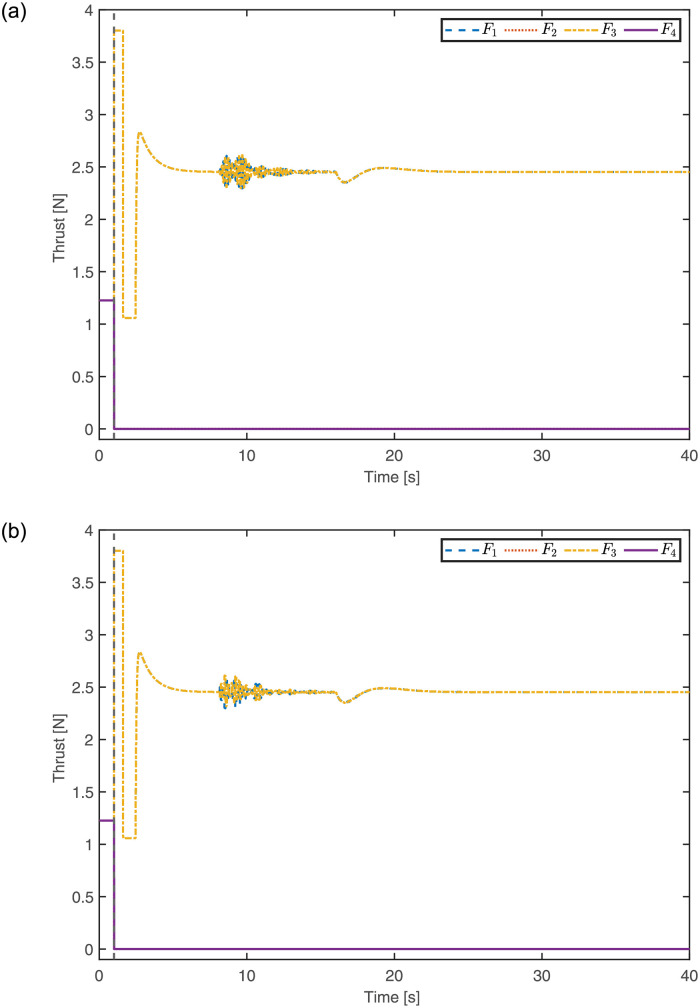
Motor thrusts of the quadrotor in case of Two opposing propellers’ failure. **(a)** Motor thrusts computed using LQI. **(b)** Motor thrusts computed using MPC.

The propellers 2 and 4 are disabled at *t* = 1s. Initially, the quadrotor uses two opposing propellers 1 and 3 (Fig 8) to gain ample and equal motor thrust to maintain the hovering state. With the fault introduced to the system in terms of propellers’ failure, new equilibrium conditions are being generated. Both controllers, LQI and MPC, are able to attain the equilibrium states with minimal effort in a timely manner. At *t* = 8s, the reference input along the *X*-axis is altered to 2m. The controllers act promptly to cater to this change and re-attain the hovering state in the presence of small transients. Another ripple effect is observed at *t* = 16s, when the height of the quadrotor drops to 0.5m. It is observed that both controllers perform well and the quadrotor successfully recovers from the failed state and achieves its hovering equilibrium solutions in both instances. By looking at Fig 8a and 8b, it can be observed that there is no significant difference in the performance of both controllers. At both occassions, the controllers are able to regain the equilibrium states robustly.

**Fig 9:**
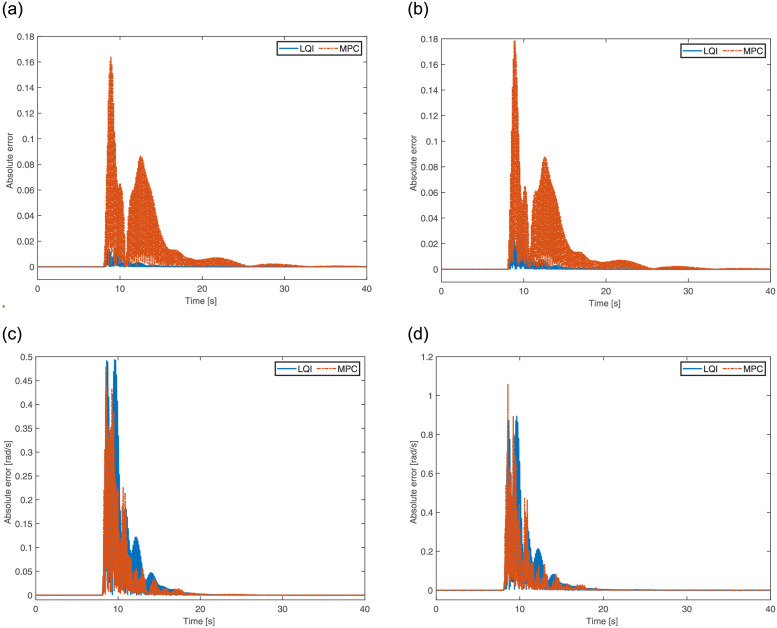
Reduced attitude error of the quadrotor for Two propellers’ failure. **(a)** Absolute error in primary axis for *η_x_*. **(b)** Absolute error in primary axis for *η_y_*. **(c)** Absolute error in angular velocity for *p*. **(d)** Absolute error in angular velocity for *q*.

The absolute error for the reduced-attitude states of the quadrotor using LQI and MPC is illustrated in Fig 9. Two propellers fail at *t* = 1s, making the quadrotor experience continuous rotation along the yaw axis. The controllers ensure that the rate of rotation remains bound to avoid any crash. The quadrotor smoothly follows the desired behavior defined using the prototype second-order system. Through the plots, not much rotation movement can be observed along the pitch-and-roll axis. Interestingly, from Fig 9a and 9b, one can see that the error in the primary axis for LQI is far lesser as compared to MPC; therefore, establishes its superiority over the latter.

## Parameter estimation for the custom quadrotor

**Fig 10:**
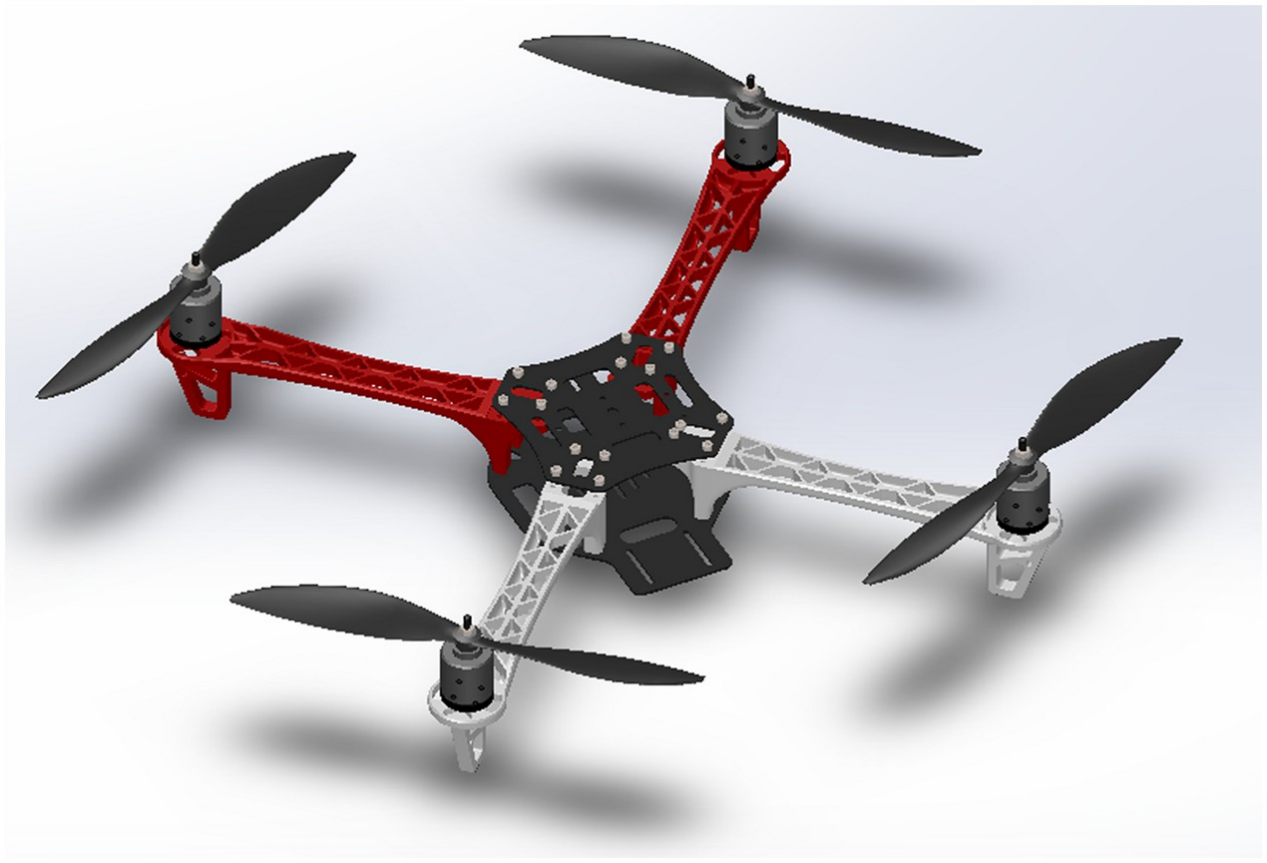
Quadrotor’s 3D CAD model.

**Fig 11:**
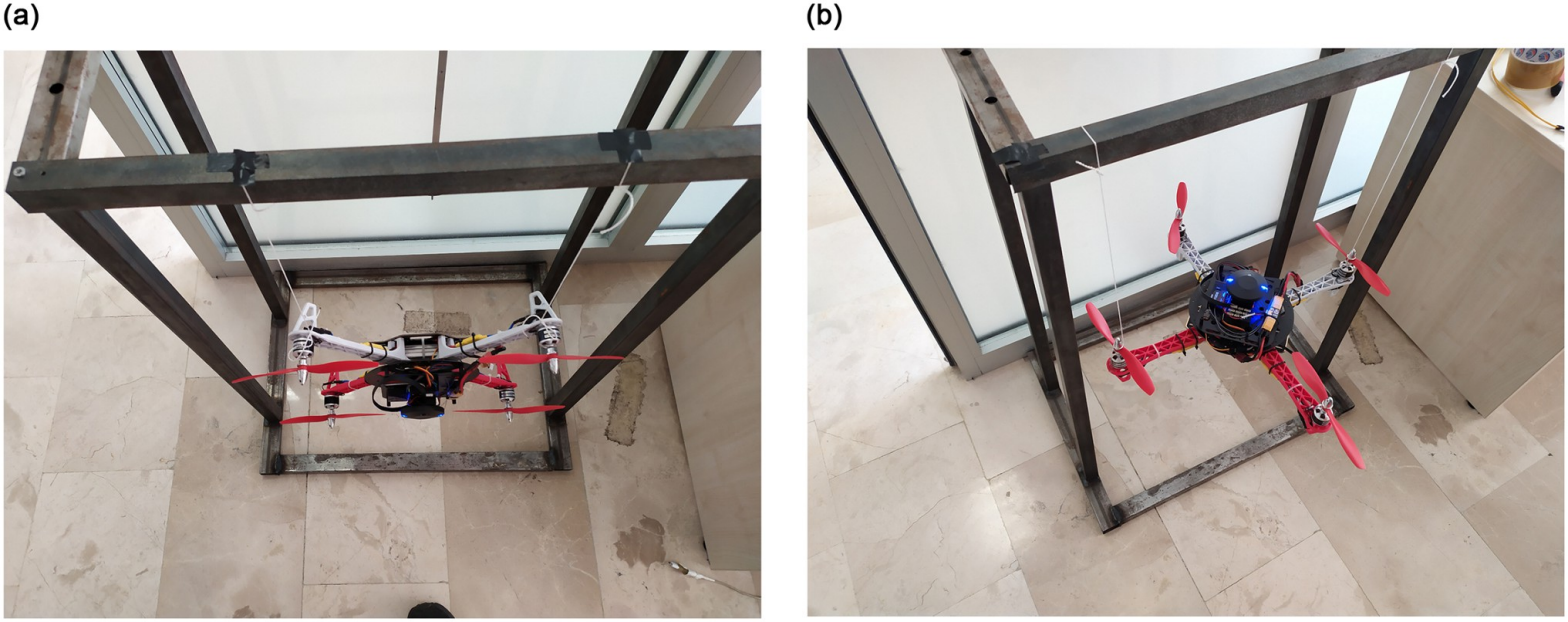
Experimental setup to perform the Bifilar pendulum experiment. **(a)** Roll test configuration. **(b)** Yaw test configuration.

A dynamic system can be represented with a set of mathematical differential equations. The best-case scenario is that the simulated system is able to replicate the behavior of an actual system to the fullest. In this work, we also tried to make such an effort by determining the unknown quadrotor’s moment of inertia. The Solidworks is utilized to obtain the quadrotor inertias on the body-fixed *X*, *Y*, and *Z* axes through a custom-made 3D model shown in Fig 10. The results are verified using the Bifiliar pendulum experiment, which deduces system inertia by oscillating the system around its own roll-and-yaw axis. The setup for the required roll-and-yaw experiments is shown in Fig 11a and 11b. The moment of inertia of the system can be estimated using the period of oscillation and the arm lengths as:

$$\begin{array}{c} I={\left[\begin{array}{c} \frac{T_{n}}{2\pi } \end{array}\right]}^{2}\frac{m\;g\;d^{2}}{l_{w}} \end{array}$$ (33)

where *T*_*n*_ is the period of oscillation, *d* is the distance between the supporting wires, and *l*_*w*_ is the wire length.

Using the data obtained through the experiment, the roll-and-pitch inertias are calculated as 0.0094kg-m^2^ and the yaw inertia is determined as 0.0175kg-m^2^. From the computer-aided design (CAD) model of the quadrotor, the roll-and-pitch inertias are determined as 0.0085kg-m^2^, whereas the inertia along the yaw is obtained as 0.0151kg-m^2^. By comparing the results, it can be confidently claimed that the parameters estimated using the Bifiliar pendulum experiment and CAD model are very close to each other.

## Conclusion

This paper proposes a novel supervisor-based active fault-tolerant control system for a quadrotor suffering from One propeller or Two opposing propellers’ failure. The supervisor module is able to sense the fault introduced to the quadrotor as propeller failure; hence, generates an appropriate excitation signal. Based on the timing and nature (one propeller, two propellers) of the excitation signal, a particular controller configuration is chosen. A multi-loop cascaded control architecture is designed to ensure robustness, stability, reference tracking, and safe landing. The outer loop is responsible for the altitude control that is achieved using PID, whereas LQI and MPC have been investigated for the reduced-attitude control in the inner loop. The failure recovery approach is tested for two different scenarios: One propeller and Two propellers’ failure. After gathering all the simulation results and performing error analysis, it has been found that the LQI has a slight edge over MPC. Additionally, the moment of inertia is estimated for the custom quadrotor using the CAD model and verified by means of the Bifiliar pendulum experiment. The estimation from these two methods supported each other.

As future work, there are several tracks that can be pursued. The control strategy proposed here can be implemented in a real-life experimental setup. Additionally, the equilibrium solutions can be optimized for multiple-objective functions, such as maximizing the flight time and (or) minimizing the energy. Another extension of this work could be the introduction of a fault diagnosis mechanism for propeller failure. In the current work, propeller failure is instantaneously detected; however, the fault detection time interval can be important in fault recovery scenarios. Lastly, future work can address the issue of dealing with two or more successive failure scenarios.
